# Seroprevalence and asymptomatic carrier status of SARS-CoV-2 in Wuhan City and other places of China

**DOI:** 10.1371/journal.pntd.0008975

**Published:** 2021-01-07

**Authors:** Shuhui Duan, Meiying Zhou, Wen Zhang, Jianwei Shen, Rui Qi, Xiangrong Qin, Hao Yu, Chuanmin Zhou, Qing Hu, Xue-Jie Yu

**Affiliations:** 1 State Key Laboratory of Virology, School of Health Sciences, Wuhan University, Wuhan, People's Republic of China; 2 Wuhan CMLabs Inc., Wuhan, People's Republic of China; 3 School of Public Health, Lanzhou University, Lanzhou, People's Republic of China; 4 Department of Clinical Laboratory, the Second Hospital of Shandong University, Jinan, People's Republic of China; WRAIR, UNITED STATES

## Abstract

Wuhan City (WH) in China was the first place to report COVID-19 in the world and the outbreak of COVID-19 was controlled in March of 2020 in WH. It is unclear what percentage of people were infected with SARS-CoV-2 and what percentage of population is carriers of SARS-CoV-2 in WH. We retrospectively analyzed the SARS-CoV-2 IgG and IgM antibody positive rates in 63,107 healthy individuals from WH and other places of China using commercial colloidal gold detection kits from March 6 to May 3, 2020. Statistical approaches were utilized to explore the difference and correlation for the seropositive rate of IgG and IgM antibody on the basis of sex, age group, geographic region and detection date. The total IgG and IgM antibody positive rate of SARS-CoV-2 was 1.68% (186/11,086) in WH, 0.59% (226/38,171) in Hubei Province without Wuhan (HB), and 0.38% (53/13,850) in the nation except for Hubei Province (CN), respectively. The IgM positive rate was 0.46% (51/11,086) in WH, 0.13% (51/38,171) in HB, and 0.07% (10/13,850) in CN. The incidence of IgM positive rates in healthy individuals increased from March 6 to May 3, 2020 in WH. Female and older age had a higher probability of becoming infected than males (OR = 1.34; 95% CI: 1.08–1.65) or younger age (OR = 2.25; 95% CI: 1.06–4.78). The seroprevalence of SARS-CoV-2 was relatively low in WH and other places of China, but it is significantly high in WH than other places of China; a large amount of asymptomatic carriers of SARS-CoV-2 existed after elimination of clinical cases of COVID-19 in Wuhan City. Therefore, SARS-CoV-2 may exist in a population without clinical cases for a long period.

## Author summary

Our study has investigated 63,107 healthy individuals from Wuhan City and other places in China to analyze the seroprevalence of SARS-CoV-2, which was correlated with geographic region, age, sex and the detection time. The seroprevalence of SARS-CoV-2 was relatively low in WH (1.68%, 186/11,086) and other places of China (0.38%, 53/13,850), but the seroprevalence is significantly higher in Wuhan City than other cities and other places in China. Moreover, the antibody positive rate was higher in regions closer to Wuhan City. The results of binary logistic regression model showed that the higher seroprevalence was observed among older individuals and females had a higher probability of being infected than males.

## Introduction

Coronavirus disease 2019 (COVID-19), caused by severe acute respiratory syndrome coronavirus 2 (SARS-CoV-2), was first reported in December of 2019 in Wuhan City, the capital of Hubei Province in China and has become a worldwide pandemic [1, 2]. As of August 4, 2020, SARS-CoV-2 has caused more than 18.1 million confirmed cases, and more than 691,000 deaths worldwide [3]. SARS-CoV-2, a novel human-infecting coronavirus, is a single-stranded positive-sense RNA (+ssRNA) virus that together with the Middle East respiratory syndrome coronavirus (MERS-CoV) and severe acute respiratory syndrome coronavirus (SARS-CoV) belongs to the *Betacoronavirus* genus [4]. SARS-CoV-2 primarily targets the human respiratory system [5, 6]. The disease spectrum caused by SARS-CoV-2 ranged from asymptomatic to death. The common symptoms of COVID-19 include fever, cough, shortness of breath, sore throat and other respiratory tract symptoms, with some patients rapidly developing acute respiratory distress syndrome (ARDS), acute respiratory failure and other serious complications such as sepsis [7–10].

Rapid and specific antibody detection could provide information to determine whether a person was infected with SARS-CoV-2 [11, 12]. The difference between IgG antibody and IgM antibody is that the presence of IgM antibody means current or recent infection with SARS-CoV-2, while IgG presence means the person was previously infected with SARS-CoV-2 [13]. The positive rate of IgG and IgM antibody during screening is an estimate of the infection rate for SARS-CoV-2 in a population.

Detection of IgM antibodies can also give a better understanding of the number of asymptomatic infections in the population at real time, as asymptomatic carriers can unwittingly transmit the SARS-CoV-2 virus person-to-person [14–16]. Currently, the antibody against SARS-CoV-2 among asymptomatic carriers remains poorly understood. Here, we conducted a retrospective study based on a large-scale serological screening for anti-SARS-CoV-2 IgG and IgM antibody among healthy individuals returning to work for a medical examination from March 6 to May 3, 2020. Moreover, the relationship between the seropositive rate and influencing factors such as sex, age distribution and geographic region was validated, which is significant for providing guidance for the resumption of work, production and school in China.

## Methods

### Ethics statement

This study was approved by the ethics committee of Wuhan University (2020YF0051). Because this public health outbreak study was a retrospective investigation, the tested persons consent for inclusion was waived.

### Data source

To assess the conditions under which asymptomatic infections occurred during the COVID-19 pandemic, we carried out a statistical analysis based on the serological survey for IgG and IgM antibody against SARS-CoV-2 among healthy individuals returning to work for a medical examination. Sera of 63,107 health persons were collected and tested from March 6 to May 3, 2020. The serology test was performed by Wuhan CMLabs Inc. using commercial colloidal gold detection kits (Innovita, Tangshan, China) with the recombinant SARS-CoV-2 N proteins as the antigens. Wuhan CMLabs Inc. is an independent medical laboratory and the physical examination data were recorded in their system by national identification numbers. The data was extracted from the system and directly packaged into a dataset only according to the detection time (from March 6 to May 3), and no sampling criteria was applied to achieve a certain expected purpose. All personal identifying information was erased except for sex, age, geographic location, and detection time.

### Analysis approaches

All statistical analyses were performed using IBM SPSS Statistics version 26.0 Data were summarized using frequency tables and figures. Pearson’s chi-square test was used to check the statistical difference in seropositive rate of both IgG and IgM antibody according to the influencing factors (sex, age group, geographic region and detection date). A binary logistic regression analysis was utilized to explore the correlation between the seropositive rate for IgG antibody and the influencing factors in Hubei Province. OR values and 95% confidence intervals of these factors were calculated. The difference was considered statistically significant with *P*<0.05.

## Results

### Seroprevalence of SARS-CoV-2 in Wuhan City, Hubei Province, and China

We analyzed IgG and IgM antibody to SARS-CoV-2 in 63,107 healthy persons from 30 provinces in Mainland China. Sera of those healthy persons were submitted by employers nationwide to test for SARS-CoV-2 antibodies. The majority of healthy persons tested were from Hubei Province (78.05%, 49,257/63,107) and the remaining 21.95% (13,850/63,107) were from other 29 provinces. The seroprevalence to SARS-CoV-2 among the healthy population of the whole nation of China was 0.74% (465/63,107). Among healthy individuals, the national IgG and IgM antibody positive rate to SARS-CoV-2 were 0.68% (432/63,107) and 0.18% (113/63,107), respectively. SARS-CoV-2 seropositive individuals were identified in 18 of 30 provinces of mainland China (Table 1). The COVID-19 epidemic was first reported in Wuhan City in Central China, which is the capital of Hubei Province. The seroprevalence of SARS-CoV-2 in Wuhan City was 1.68% (186/11,086). Considering that the high serum antibody positive rate in Wuhan City might raise the serum antibody positive rate in Hubei Province and the whole country due to relatively large amount of samples from Wuhan City, we analyzed the antibody positive rate for Hubei Province except for Wuhan City, the nation of China without Hubei Province, respectively. When the data of Wuhan City was deducted from Hubei Province and the data of Hubei Province was deducted from the national data, the corrected seroprevalence of SARS-CoV-2 in Hubei Province and China were 0.59% (226/38,171) and 0.38% (53/13,850), respectively, suggesting that the seroprevalence of SARS-CoV-2 is significantly higher in Wuhan City than that of other cities of Hubei Province and other places in China.

**Table 1:** The total IgG and IgM antibody positive rate of SARS-CoV-2 in different provinces in mainland China.

	Number of persons tested	IgG positive persons	IgM positive persons	Total antibody positive persons	IgG positive rate (%)	IgM positive rate (%)	Total antibody positive rate (%)
Hubei	49,257	382	102	412	0.78	0.21	0.84
Henan	3,956	18	3	19	0.46	0.08	0.48
Hunan	1,312	5	1	5	0.38	0.08	0.38
Anhui	844	2	2	3	0.24	0.24	0.36
Sichuan	803	5	1	5	0.62	0.12	0.62
Shaanxi	790	2	0	2	0.25	0.00	0.25
Jiangxi	708	6	2	6	0.85	0.28	0.85
Jiangsu	683	1	0	1	0.15	0.00	0.15
Shandong	582	1	0	1	0.17	0.00	0.17
Guizhou	573	1	0	1	0.17	0.00	0.17
Hebei	434	0	1	1	0.00	0.23	0.23
Gansu	375	1	0	1	0.27	0.00	0.27
Chongqing	359	0	0	0	0.00	0.00	0.00
Yunnan	321	1	0	1	0.31	0.00	0.31
Shanxi	309	0	0	0	0.00	0.00	0.00
Other provinces	1,801	7	1	7	0.39	0.06	0.39
Total	63,107	432	113	465	0.68	0.18	0.74

Note: the samples of provinces with less than 300 healthy persons each were all summarized together as other provinces because the small sample size may generate bias on seroprevalence.

**Fig 1:**
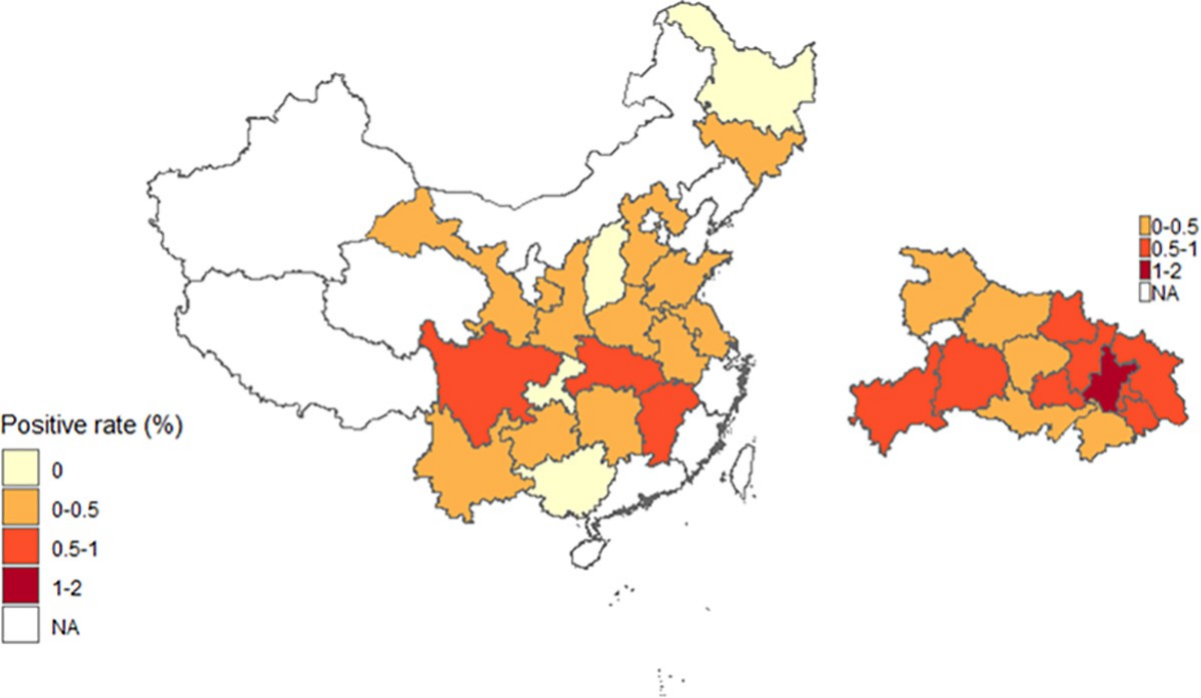
The total IgG and IgM antibody positive rate of SARS-CoV-2 in Hubei Province and other provinces with sample size >300 persons in mainland China. Asymptomatic carriers of SARS-CoV-2 in Wuhan City, Hubei Province, and China.

Within Hubei Province, individuals seropositive to SARS-CoV-2 were identified in all cities except for the Shennongjia area, which had a small sample size (Table 2). The seroprevalence is highest in Wuhan City, and the cities bordering Wuhan City have a higher seroprevalence than cities more distant from it (Fig 1).

**Table 2:** Single factor analysis of seroprevalence in Hubei Province.

	Number of tested persons	Seroprevalence (%)		χ^2^		*P*-value	
		IgG antibody	IgM antibody	IgG seropositive	IgM seropositive	IgG seropositive	IgM seropositive
Total	49,257	0.78	0.21				
Sexes				12.92	0.38	**<0.001**	0.538
Male	35,628	0.69	0.20				
Female	13,629	1.01	0.23				
Age groups				79.61*	51.99*	**<10**^−20^*	**<10**^−12^*
age≤20	1,324	0.60	0				
20<age≤30	23,444	0.57	0.13				
30<age≤40	18,178	0.74	0.20				
40<age≤50	3,778	1.22	0.34				
age>50	2,533	2.37	0.87				
Geographic regions				161.12	50.45	**<10**^**−25**^	**<10**^**−4**^
Wuhan	11,086	1.61	0.46				
Ezhou	3,685	0.84	0.14				
Enshi	1,066	0.56	0.09				
Huanggang	8,387	0.51	0.14				
Huangshi	2,268	0.53	0.22				
Jingmen	1,759	0.23	0.00				
Jingzhou	3,048	0.46	0.07				
Yichang	1,541	0.45	0.26				
Shiyan	2,179	0.41	0.09				
Xiaogan	5,276	0.91	0.15				
Xiangyang	4,484	0.13	0.09				
Xianning	1,134	0.35	0.09				
Qianjiang	440	0.45	0.23				
Suizhou	946	0.42	0.21				
Tianmen	1,038	0.96	0.19				
Xiantao	893	0.34	0.22				
Shennongjia	27	0.00	0.00				

Note: statistically significant variables are indicated by bold typing.

*: Chi-square for trend.

People with IgM antibody positive to SARS-CoV-2 were found in 8 of 30 provinces of mainland China (Table 1). The IgM positive rate is significantly higher in Wuhan City (0.46%, 51/11,086) than in other places of Hubei Province (0.13%, 51/38,171, *P* = 1.94×10^−5^) and other places of China (0.08%, 11/13,850, *P* = 0.968). All individuals tested for SARS-CoV-2 antibody in this study were healthy and without clinical symptoms of COVID-19. These results indicate that asymptomatic carriers of SARS-CoV-2 exists in large areas in China and that there are many people who were asymptomatic carriers of SARS-CoV-2 in Wuhan City.

### Correlation between antibody positive rate and influencing factors in Hubei Province

The correlation between the positive rate of antibodies and the influencing factors in Hubei Province was analyzed via a single factor analysis by Pearson chi-square (χ^2^) tests (Table 2). Both IgG antibody (χ^2^ = 79.61, *P*<10^−20^) and IgM antibody (χ^2^ = 51.99, *P*<10^−12^) had significant correlation between their serum positive rate and age group by the chi-square test for trend. Moreover, similar results can be seen for regional factors for the IgG antibody positive rate (χ^2^ = 161.12, *P*<10^−25^) and IgM antibody positive rate (χ^2^ = 50.45, *P*<10^−4^).

A binary logistic regression was then performed to identify the correlation between the IgG antibody positive rate and sex, age groups and geographic regions. The results of the model showed that sex, ages under 20 or over 50 years old, and most of the geographic regions (cities) in Hubei Province were all related to IgG antibody positive rate (*P*<0.05). Females showed a higher probability of becoming infected (OR = 1.34; 95% CI: 1.08–1.65). Considering age, there were 2.25 times more seropositive individuals >50 years old (2.37%) than individuals who were less than 20 years old (0.60%) (OR = 2.25; 95% CI: 1.06–4.78). The influencing factor of geographic region was highly significant. This finding indicated that the probability of an individual having been infected with SARS-CoV-2 in Xiangyang City (0.13%) or Jingmeng City (0.23%) was 0.1 times or 0.17 times less than an individual in Wuhan City (Table 3).

**Table 3:** Binary logistic regression of the influencing factors for IgG antibody positive rate in Hubei Province.

Variables	IgG antibody positive rate (%)	*P*-value	OR	95% CI
Sexes				
male*	0.69	-	-	-
female	1.01	**0.007**	1.34	1.08–1.65
Age groups				
age≤20*	0.60	**-**	-	-
20<age≤30	0.57	0.686	0.86	0.42–1.77
30<age≤40	0.74	0.973	1.01	0.49–2.08
40<age≤50	1.22	0.436	1.35	0.63–2.90
age>50	2.37	**0.035**	2.25	1.06–4.78
Geographic regions				
Wuhan*	1.61	**-**	-	-
Ezhou	0.84	**0.001**	0.53	0.36–0.77
Enshi	0.56	**0.037**	0.42	0.18–0.95
Huanggang	0.51	**<10**^**−7**^	0.39	0.28–0.55
Huangshi	0.53	**0.002**	0.40	0.22–0.72
Jingmen	0.23	**<0.001**	0.17	0.06–0.46
Jingzhou	0.46	**<0.001**	0.35	0.20–0.62
Yichang	0.45	**0.007**	0.35	0.16–0.75
Shiyan	0.41	**0.001**	0.32	0.16–0.64
Xiaogan	0.91	**0.026**	0.69	0.49–0.96
Xiangyang	0.13	**<10**^**−7**^	0.10	0.04–0.22
Xianning	0.35	**0.014**	0.29	0.11–0.78
Qianjiang	0.45	0.151	0.36	0.09–1.46
Suizhou	0.42	**0.019**	0.30	0.11–0.82
Tianmen	0.96	0.353	0.74	0.39–1.40
Xiantao	0.34	**0.021**	0.26	0.08–0.81
Shennongjia	0.00	0.998	4.28×10^−8^	0.00

Note:

*Reference category, OR = Odds ratio, 95% CI: 95% Confidence Interval. Statistically significant variables are indicated by bold typing.

### SARS-CoV-2 seroprevalence between sexes and among age groups

**Fig 2:**
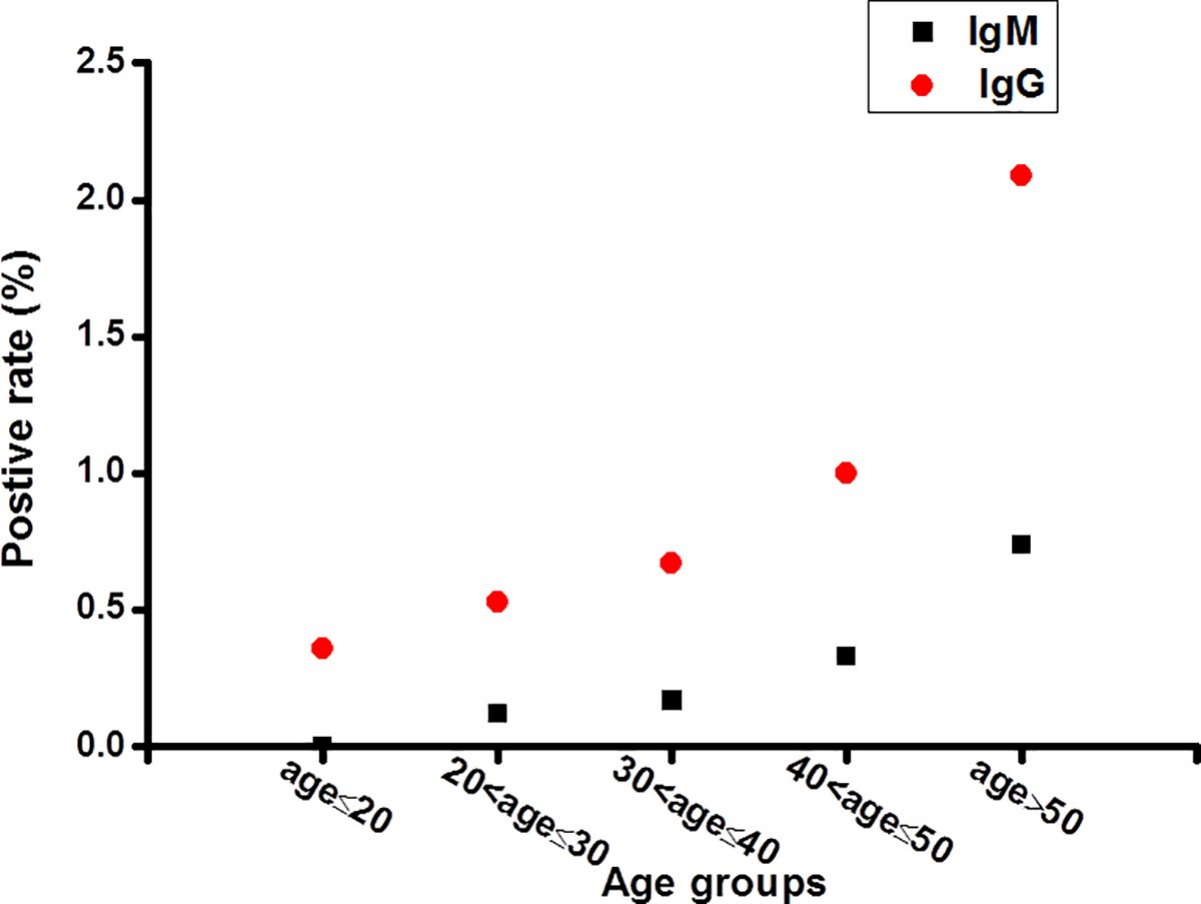
IgG and IgM positive rates of SARS-CoV-2 among age groups.

Among 63,107 tested individuals, there were 46,143 males and 16,964 females (male-to-female ratio: 2.72:1). The median age of them was 30 years old (range: 17–63 years old). The immune response of the tested individuals was different by sex and age. Females had greater odds of being IgG positive than males (0.91% and 0.60%, respectively; χ^2^ = 17.01, *P* < 10^−4^), while the rate of being IgM positive for females and males was 0.22% and 0.16%, respectively (χ^2^ = 2.62, *P* = 0.105) (Table 4). It can be concluded that women were more susceptible than men. We analyzed further the proportion of male and female in each age group, and it showed that female proportion in every age group was less than male (18.85% - 32.26%). At the same time, with the increase of age and female proportion, the positive rate of antibodies increased among these age groups. This finding was consistent with what was previously reported [17], suggesting that females have higher levels of activation of immune cells, leading to higher levels of antibodies against SARS-CoV-2 produced in females than in males. Moreover, the highest seroprevalence was observed among people >50 years old (2.09% for IgG positive, 0.74% for IgM positive), while those between 41–50 years old had a 1.00% IgG positive rate and 0.33% IgM positive rate and those less than 20 years old had a 0.36% IgG positive rate and no IgM positives. As shown in Fig 2, antibodies against SARS-CoV-2 increased with age, indicating that the odds of being infected increased. These differences were statistically significant (χ^2^ = 107.67 for IgG positive rate, P<10^−21^ and χ^2^ = 70.34 for IgM positive rate, *P***<**10^−13^) (Fig 2 and Table 4).

**Table 4:** Seroprevalence according to sexes and age groups.

	Number of tested persons	IgG		IgM	
		Positive persons	Positive rate (%)	Positive persons	Positive rate (%)
Sexes					
Male	46,143	278	0.60	75	0.16
Female	16,964	154	0.91	38	0.22
χ^2^		17.01		2.62	
*P*-value		**<10**^**−4**^		0.105	
Age groups					
age≤20	2,499	9	0.36	0	0
20<age≤30	29,428	156	0.53	34	0.12
30<age≤40	23,099	154	0.67	40	0.17
40<age≤50	5,111	51	1.00	17	0.33
age>50	2,970	62	2.09	22	0.74
χ^2^		107.67		70.34	
*P*-value		**<10**^**−21**^		**<10**^**−13**^	

Note: statistically significant variables are indicated by bold typing.

**Fig 3:**
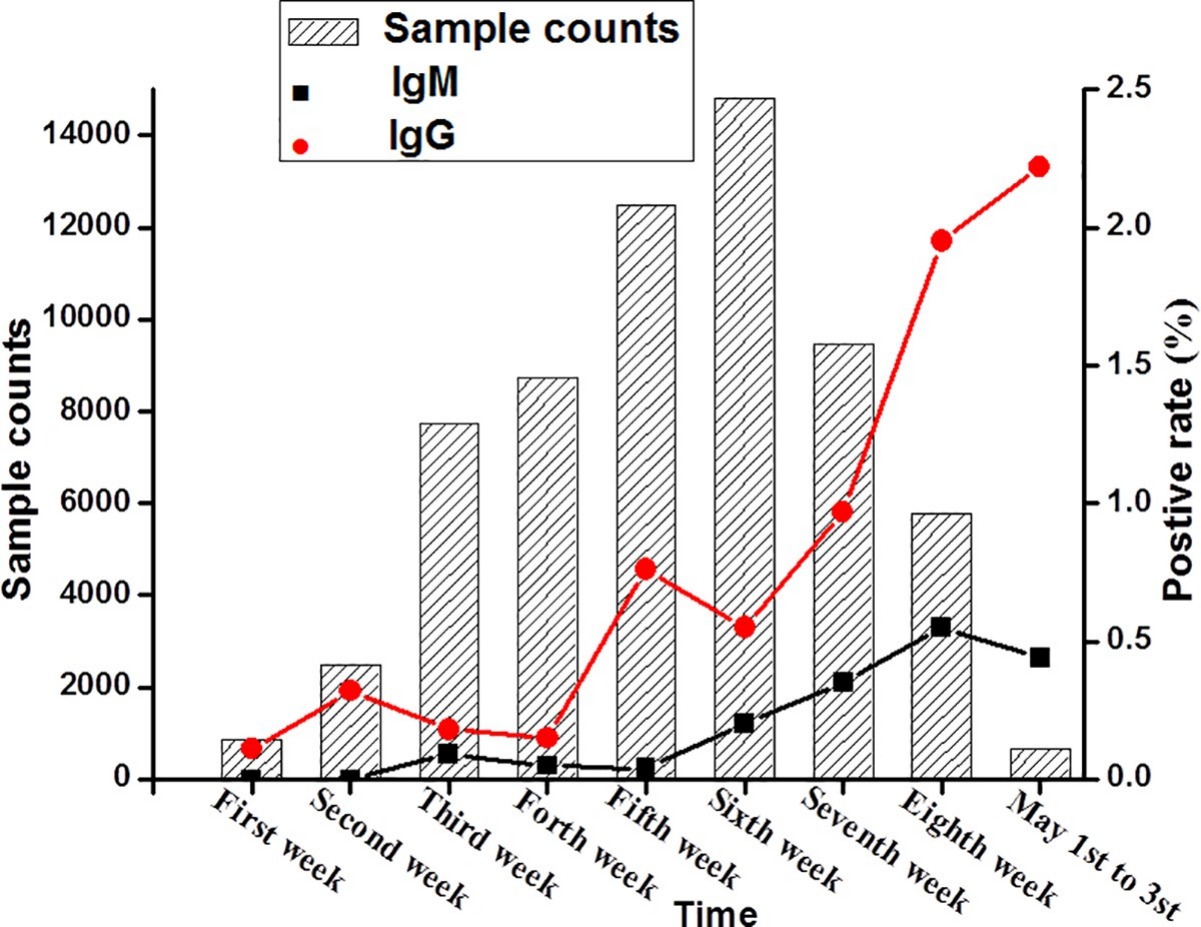
Antibody positive rates changed by weeks from March 6 to May 3, 2020.

The tested sample numbers, positive rates for IgG antibody and IgM antibody were stacked to show the seroprevalence by weeks from March 6 to May 3 (Fig 3). As Fig 3 shows, the positive rate of IgG antibody fluctuated greatly, while IgM fluctuation was relatively small during the two months, but IgM positive rate had been increased steadily from March 6 to May 3, 2020 (range: 0–1.00%). Overall, the IgG antibody positive rate was higher than the IgM antibody positive rate for the same tested individuals (Fig 3).

## Discussion

The epidemic of COVID-19 was first reported in Wuhan City in central China on December 12, 2019 and quickly spread to other places in China by the middle of January of 2020. A 76-day lockdown of Wuhan City and Hubei Province from January 23, 2020 had effectively restricted the spread of SARS-CoV-2 to other places in China, leading to the majority of COVID-19 cases occurring in Wuhan City and its adjacent cities in Hubei Province. As of May 19, 2020, China reported 78,241 COVID-19 cases with 64.3% (50,340 cases) from Wuhan City and 22.7% (17,795 cases) from other cities adjacent to Wuhan City in Hubei Province. The community transmission of COVID-19 was effectively controlled by strict stay-at-home orders in affected areas. We analyzed the seroprevalence of SARS-CoV-2 in healthy individuals from Wuhan City and other places in China. Consistent with a large number of COVID-19 cases having occurred in Wuhan, the seroprevalence of SARS-CoV-2 is significantly higher in Wuhan City than other regions of Hubei Province and other places in China. Within Hubei Province, the seroprevalence of SARS-CoV-2 was higher in cities which are closer geographically to Wuhan City than in cities distant from it, and the seroprevalence of SARS-CoV-2 was higher in provinces closer to Hubei Province such as Jiangxi, Henan, Hunan and Anhui provinces, suggesting the spread of SARS-CoV-2 from Wuhan City to its surrounding areas.

Our study has investigated a large amount of samples based on the healthy individuals returning to work from Wuhan City, other cities of Hubei Province, and other places in China. Although the sample size was large, there might be bias since the date was obtained from healthy individuals rather than total population. Based on 1.68% seroprevalence and population of 10 millions in Wuhan City, we estimated that 168,000 people were infected with SARS-CoV-2 during the COVID-19 epidemic in the city. Wuhan City has reported 50,340 hospitalized COVID-19 patients as of May 19, 2020, indicating that only one third SARS-CoV-2 infected persons need to be hospitalized and at least 2/3 infected persons were asymptomatic in the city. According to the IgM positive rate (0.46%, 51/11.086) in Wuhan City, we estimate thousands of people had been infected asymptomatically in a period of 2 months from March 6 to May 3 of 2020 based on the population in the city. On June 3 the Wuhan Municipal Health Commission reported that the asymptomatic infection rate was 0.303/10,000 based on nucleic acid test of 9.9 million people in Wuhan City from May 14 to June 1. The asymptomatic infection rate based on IgM positive rate in this study was hundreds times of that based on the nucleic acid test. The discrepancy between our result and the nucleic acid test could be caused by difference in sample collection time and the sensitivity of the methods. The samples in our study were collected mainly from March to April, which was 2–3 months early than the samples tested by nucleic acid. The serology test is more sensitive than PCR test of nucleic acid. The early report indicated that less than 40% patients were positive by PCR test [18] and the sensitivity of PCR test for screen of asymptomatic carrier should be further decreased because of combination of multiple persons’ samples together to reduce the cost. Another possibility for the much high IgM positive rate than PCR is that IgM positive may be overestimated with colloidal kits. The specificity and sensitivity of colloidal gold kits ranged from 90.6% to 96.2% and 71.1% to 88.7%, respectively [13, 19]. The question is why so many people were IgM positive to SARS-CoV-2, but very few persons got sick. Our hypothesis is that strains that cause asymptomatic infection in Wuhan City and other places in China are attenuated viral strains. China has actively searched for COVID-19 patients and the symptomatic patients were hospitalized, which has restricted the ability of the viral strains that caused severe disease from spreading to new hosts and was eventually eliminated. However, it is difficult to identify the avirulent SARS-CoV-2 strains that cause asymptomatic infection in the population. Avirulent SARS-CoV-2 strains may still cause symptoms in extremely susceptible individuals and it may also revert to a highly virulent strain to reignite the epidemic of COVID-19 in China. Therefore, we need to pay attention to and identify COVID-19 patients in Wuhan City and other places in China.

Our study indicated that the seroprevalence of the normal population was significantly correlated with age, sex and geographic region, and at the same time the positive rate would change with the passage of detection time. In order to further assess the relationship between the IgG antibody positive rate and influencing factors, such as sex, age, and geographic regions, we identified that the IgG antibody positive rate was well correlated with those influencing factors in Hubei Province (*P*<0.05). Moreover, the results of binary logistic regression model showed that females had a higher probability of become infected, and older individuals had a higher probability of become infected than younger ones.

We conclude that the seroprevalence of SARS-CoV-2 is relatively low in Wuhan City and other places of China, but it is significantly higher in Wuhan City than in other places of China and a large amount of asymptomatic carriers of SARS-CoV-2 existed after elimination clinical cases of COVID-19 in Wuhan City. Therefore, SARS-CoV-2 may exist in a population without clinical cases.
